# Supplementary material for: Climate Change, Drought and Rural Suicide in New South Wales, Australia: Future Impact Scenario Projections to 2099
Source: Int J Environ Res Public Health. 2022 Jun 27;19(13):7855. doi: 10.3390/ijerph19137855 (PMC9266200; doi:10.3390/ijerph19137855)
Supplement: Supplementary file 1 [file ijerph-19-07855-s001.zip › ijerph-1701945-SI.pdf]

Supplementary Information for Climate Change, Drought and Rural Suicide in New South Wales, Australia: Future Impact Scenario Projections to 2099

Potential confounders: seasonal cycle and temperature anomalies

The main statistical model focused on the drought index but also adjusted for other well-known effects on suicide rates, including increased suicide risks during spring (Durkheim 1897, 1951) and times of unusually high maximum temperatures (Mullins 2019). These variables were included to account for potential confounding because a) droughts may be related to seasonal rainfall patterns and b) droughts may correlate with higher than average temperatures due to lack of cloud cover in summer. The resulting drought effect estimates are therefore considered independent of these other important variables. The seasonal cycle estimated from the final model used a cyclic spline with 4 degrees of freedom and is shown in Figure S1 below. Monthly maximum temperature anomalies were calculated as the difference between each month's temperature and the long-term average for that month. The estimated relative risk (RR) for monthly maximum temperature anomaly was 1.02 (95%CI 1.01, 1.03) per degree C.

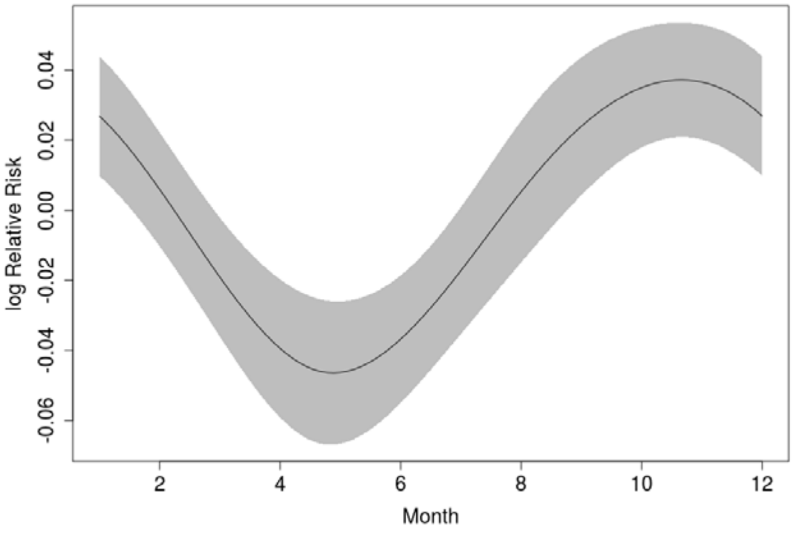

Figure S1: the seasonal cycle estimated in the final model using a cyclic cubic spline showing the well-known peak in suicides during springtime.

All estimated suicide deaths attributable to full drought

Table S1: Attributable number and fraction of suicide deaths due to full drought (drought index above 6 months) for each age/sex category; the ACCESS1.0 model had maximum consensus with the other models for many regions, the GFDL-ESM2M model predicts hotter and drier conditions for many regions and the NorESM1-M model predicts a lower warming, wetter scenario. The model was not penalized and no spline was used.

Sex	Age group	Scenario	GCM	RCP	Attributable number	Lower 2.5%	Upper 97.5%	Baseline Annual N	Attributable fraction (%)
Males	10_29	historical	historical	historical	0.31	-0.05	0.63	33.26	0.93

Males	10_29	access1p0_rcp4p5	access1p0	rcp4p5	0.44	-0.11	0.90	33.26	1.33
Males	10_29	access1p0_rcp8p5	access1p0	rcp8p5	0.33	-0.08	0.67	33.26	0.99
Males	10_29	gfdl_esm2m_rcp4p5	gfdl_esm2m	rcp4p5	0.60	-0.11	1.23	33.26	1.81
Males	10_29	gfdl_esm2m_rcp8p5	gfdl_esm2m	rcp8p5	0.57	-0.10	1.16	33.26	1.71
Males	10_29	noresm1_m_rcp4p5	noresm1_m	rcp4p5	0.36	-0.05	0.74	33.26	1.08
Males	10_29	noresm1_m_rcp8p5	noresm1_m	rcp8p5	0.31	-0.04	0.64	33.26	0.94
Males	30_49	historical	historical	historical	0.82	0.45	1.15	45.24	1.81
Males	30_49	access1p0_rcp4p5	access1p0	rcp4p5	1.13	0.59	1.59	45.24	2.50
Males	30_49	access1p0_rcp8p5	access1p0	rcp8p5	0.84	0.44	1.18	45.24	1.85
Males	30_49	gfdl_esm2m_rcp4p5	gfdl_esm2m	rcp4p5	1.51	0.84	2.13	45.24	3.34
Males	30_49	gfdl_esm2m_rcp8p5	gfdl_esm2m	rcp8p5	1.43	0.79	2.01	45.24	3.16
Males	30_49	noresm1_m_rcp4p5	noresm1_m	rcp4p5	0.94	0.53	1.32	45.24	2.07
Males	30_49	noresm1_m_rcp8p5	noresm1_m	rcp8p5	0.82	0.46	1.16	45.24	1.81
Males	50plus	historical	historical	historical	-0.06	-0.53	0.36	41.63	-0.15
Males	50plus	access1p0_rcp4p5	access1p0	rcp4p5	-0.09	-0.79	0.50	41.63	-0.21
Males	50plus	access1p0_rcp8p5	access1p0	rcp8p5	-0.07	-0.59	0.38	41.63	-0.16
Males	50plus	gfdl_esm2m_rcp4p5	gfdl_esm2m	rcp4p5	-0.12	-1.04	0.70	41.63	-0.29
Males	50plus	gfdl_esm2m_rcp8p5	gfdl_esm2m	rcp8p5	-0.11	-0.98	0.66	41.63	-0.28
Males	50plus	noresm1_m_rcp4p5	noresm1_m	rcp4p5	-0.07	-0.60	0.42	41.63	-0.17
Males	50plus	noresm1_m_rcp8p5	noresm1_m	rcp8p5	-0.06	-0.53	0.37	41.63	-0.15
Females	10_29	historical	historical	historical	-0.05	-0.26	0.10	6.18	-0.88
Females	10_29	access1p0_rcp4p5	access1p0	rcp4p5	-0.08	-0.38	0.14	6.18	-1.24
Females	10_29	access1p0_rcp8p5	access1p0	rcp8p5	-0.06	-0.28	0.10	6.18	-0.91
Females	10_29	gfdl_esm2m_rcp4p5	gfdl_esm2m	rcp4p5	-0.11	-0.51	0.19	6.18	-1.72
Females	10_29	gfdl_esm2m_rcp8p5	gfdl_esm2m	rcp8p5	-0.10	-0.48	0.18	6.18	-1.62
Females	10_29	noresm1_m_rcp4p5	noresm1_m	rcp4p5	-0.06	-0.30	0.12	6.18	-1.05
Females	10_29	noresm1_m_rcp8p5	noresm1_m	rcp8p5	-0.05	-0.25	0.10	6.18	-0.87
Females	30_49	historical	historical	historical	-0.29	-0.61	-0.03	10.21	-2.79
Females	30_49	access1p0_rcp4p5	access1p0	rcp4p5	-0.39	-0.86	-0.04	10.21	-3.84
Females	30_49	access1p0_rcp8p5	access1p0	rcp8p5	-0.29	-0.63	-0.03	10.21	-2.82
Females	30_49	gfdl_esm2m_rcp4p5	gfdl_esm2m	rcp4p5	-0.53	-1.14	-0.05	10.21	-5.22
Females	30_49	gfdl_esm2m_rcp8p5	gfdl_esm2m	rcp8p5	-0.50	-1.06	-0.05	10.21	-4.86
Females	30_49	noresm1_m_rcp4p5	noresm1_m	rcp4p5	-0.32	-0.66	-0.03	10.21	-3.09
Females	30_49	noresm1_m_rcp8p5	noresm1_m	rcp8p5	-0.28	-0.59	-0.03	10.21	-2.77
Females	50plus	historical	historical	historical	-0.61	-1.01	-0.29	9.74	-6.26
Females	50plus	access1p0_rcp4p5	access1p0	rcp4p5	-0.83	-1.42	-0.40	9.74	-8.54
Females	50plus	access1p0_rcp8p5	access1p0	rcp8p5	-0.62	-1.05	-0.30	9.74	-6.36
Females	50plus	gfdl_esm2m_rcp4p5	gfdl_esm2m	rcp4p5	-1.12	-1.86	-0.53	9.74	-11.46
Females	50plus	gfdl_esm2m_rcp8p5	gfdl_esm2m	rcp8p5	-1.05	-1.74	-0.50	9.74	-10.76
Females	50plus	noresm1_m_rcp4p5	noresm1_m	rcp4p5	-0.70	-1.15	-0.33	9.74	-7.18
Females	50plus	noresm1_m_rcp8p5	noresm1_m	rcp8p5	-0.61	-1.00	-0.29	9.74	-6.24

All estimated suicide deaths attributable to any month in drought

Table S2: Attributable number and fraction of suicide deaths due to all levels of drought index (i.e. based on all months where drought index > 0) by each age/sex category; the ACCESS1.0 model had maximum consensus with the other models for many regions, the GFDL-ESM2M model predicts hotter and drier conditions for many regions and the NorESM1-M model predicts a lower warming, wetter scenario.

Sex	Age	Scenario	GCM	RCP	Attributable	Lower	Upper	Baseline	Attributable

	group				number	2.5%	97.5%	Annual N	fraction (%)
Males	10_29	historical	historical	historical	0.88	-0.15	1.84	33.26	2.65
Males	10_29	access1p0_rcp4p5	access1p0	rcp4p5	0.99	-0.24	2.05	33.26	2.97
Males	10_29	access1p0_rcp8p5	access1p0	rcp8p5	0.92	-0.23	1.91	33.26	2.77
Males	10_29	gfdl_esm2m_rcp4p5	gfdl_esm2m	rcp4p5	1.15	-0.20	2.37	33.26	3.45
Males	10_29	gfdl_esm2m_rcp8p5	gfdl_esm2m	rcp8p5	1.17	-0.20	2.42	33.26	3.51
Males	10_29	noresm1_m_rcp4p5	noresm1_m	rcp4p5	0.90	-0.13	1.89	33.26	2.71
Males	10_29	noresm1_m_rcp8p5	noresm1_m	rcp8p5	0.81	-0.11	1.70	33.26	2.45
Males	30_49	historical	historical	historical	2.32	1.26	3.32	45.24	5.13
Males	30_49	access1p0_rcp4p5	access1p0	rcp4p5	2.59	1.34	3.68	45.24	5.72
Males	30_49	access1p0_rcp8p5	access1p0	rcp8p5	2.41	1.24	3.44	45.24	5.33
Males	30_49	gfdl_esm2m_rcp4p5	gfdl_esm2m	rcp4p5	2.97	1.62	4.22	45.24	6.56
Males	30_49	gfdl_esm2m_rcp8p5	gfdl_esm2m	rcp8p5	3.04	1.65	4.32	45.24	6.71
Males	30_49	noresm1_m_rcp4p5	noresm1_m	rcp4p5	2.38	1.32	3.40	45.24	5.25
Males	30_49	noresm1_m_rcp8p5	noresm1_m	rcp8p5	2.14	1.19	3.07	45.24	4.74
Males	50plus	historical	historical	historical	-0.17	-1.46	1.02	41.63	-0.42
Males	50plus	access1p0_rcp4p5	access1p0	rcp4p5	-0.20	-1.73	1.14	41.63	-0.47
Males	50plus	access1p0_rcp8p5	access1p0	rcp8p5	-0.18	-1.60	1.06	41.63	-0.44
Males	50plus	gfdl_esm2m_rcp4p5	gfdl_esm2m	rcp4p5	-0.23	-1.94	1.34	41.63	-0.55
Males	50plus	gfdl_esm2m_rcp8p5	gfdl_esm2m	rcp8p5	-0.23	-1.97	1.36	41.63	-0.56
Males	50plus	noresm1_m_rcp4p5	noresm1_m	rcp4p5	-0.18	-1.47	1.06	41.63	-0.43
Males	50plus	noresm1_m_rcp8p5	noresm1_m	rcp8p5	-0.16	-1.32	0.96	41.63	-0.39
Females	10_29	historical	historical	historical	-0.15	-0.68	0.28	6.18	-2.44
Females	10_29	access1p0_rcp4p5	access1p0	rcp4p5	-0.17	-0.80	0.31	6.18	-2.70
Females	10_29	access1p0_rcp8p5	access1p0	rcp8p5	-0.15	-0.73	0.29	6.18	-2.48
Females	10_29	gfdl_esm2m_rcp4p5	gfdl_esm2m	rcp4p5	-0.20	-0.91	0.37	6.18	-3.20
Females	10_29	gfdl_esm2m_rcp8p5	gfdl_esm2m	rcp8p5	-0.20	-0.92	0.37	6.18	-3.23
Females	10_29	noresm1_m_rcp4p5	noresm1_m	rcp4p5	-0.15	-0.69	0.29	6.18	-2.51
Females	10_29	noresm1_m_rcp8p5	noresm1_m	rcp8p5	-0.14	-0.61	0.26	6.18	-2.23
Females	30_49	historical	historical	historical	-0.74	-1.53	-0.08	10.21	-7.29
Females	30_49	access1p0_rcp4p5	access1p0	rcp4p5	-0.83	-1.78	-0.09	10.21	-8.17
Females	30_49	access1p0_rcp8p5	access1p0	rcp8p5	-0.75	-1.60	-0.08	10.21	-7.39
Females	30_49	gfdl_esm2m_rcp4p5	gfdl_esm2m	rcp4p5	-0.97	-2.02	-0.10	10.21	-9.52
Females	30_49	gfdl_esm2m_rcp8p5	gfdl_esm2m	rcp8p5	-0.98	-2.03	-0.10	10.21	-9.59
Females	30_49	noresm1_m_rcp4p5	noresm1_m	rcp4p5	-0.76	-1.54	-0.07	10.21	-7.43
Females	30_49	noresm1_m_rcp8p5	noresm1_m	rcp8p5	-0.68	-1.38	-0.07	10.21	-6.68
Females	50plus	historical	historical	historical	-1.54	-2.46	-0.76	9.74	-15.82
Females	50plus	access1p0_rcp4p5	access1p0	rcp4p5	-1.73	-2.85	-0.85	9.74	-17.74
Females	50plus	access1p0_rcp8p5	access1p0	rcp8p5	-1.59	-2.60	-0.79	9.74	-16.32
Females	50plus	gfdl_esm2m_rcp4p5	gfdl_esm2m	rcp4p5	-2.03	-3.30	-0.99	9.74	-20.86
Females	50plus	gfdl_esm2m_rcp8p5	gfdl_esm2m	rcp8p5	-2.06	-3.32	-1.01	9.74	-21.13
Females	50plus	noresm1_m_rcp4p5	noresm1_m	rcp4p5	-1.59	-2.52	-0.77	9.74	-16.30
Females	50plus	noresm1_m_rcp8p5	noresm1_m	rcp8p5	-1.41	-2.24	-0.69	9.74	-14.53

Temporal change in the attributable number (AN) by quarter-centuries

The main text reports the attributable number (AN) per annum in two broad periods (1971–1999 *vs.* 2000–2099). As an alternative to show temporal changes in the AN by

sub-periods, Figure S2 shows that for males (and 95% CI) for quarter-centuries (25-year periods) and Figure S3 shows the results for females. This demonstrates differences in the estimated AN changes over time and differs between climate change scenarios. The apparent declining impact in the driest scenario (GFDL-ESM2M) where initial drought impacts are high but then ease in the later part of the century is explained by the adaptation effect captured by the 30-year lookback period. Specifically, perceptions of dry months at the end of a dry 30-year period will not be as severe as those of dry months after a relatively wetter 30-year period.

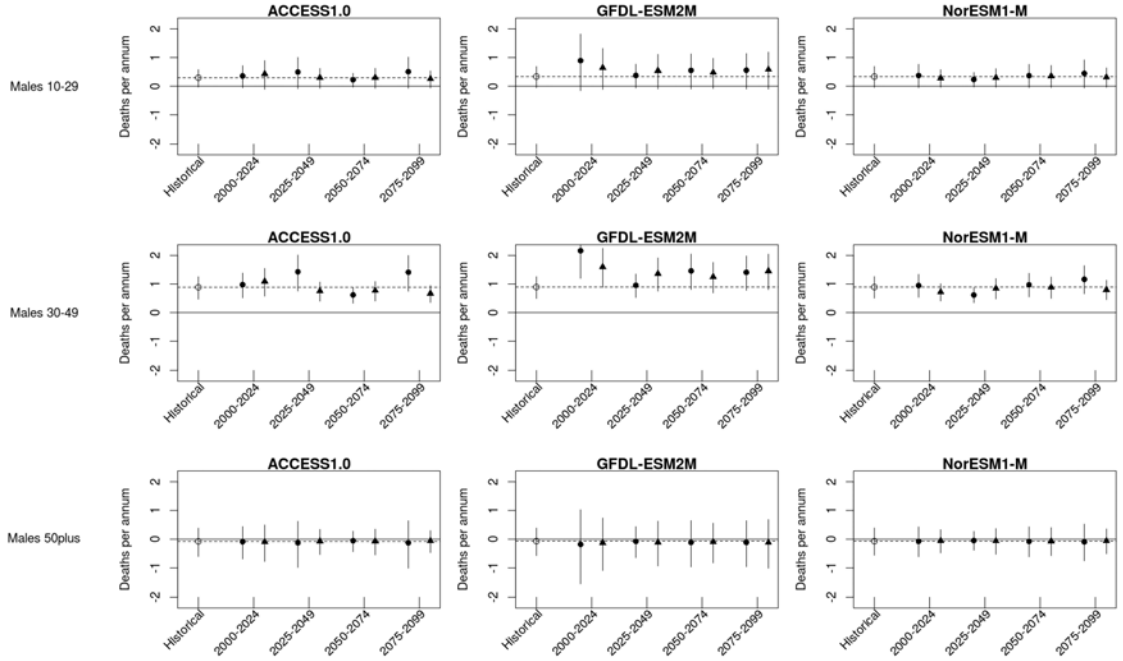

Figure S2: ANs (and 95% CI) among males for quarter-centuries (25 year periods) compared to historical rates (1975–1999). The righthand triangular dots in each pair are RCP8.5 (high emissions scenario) while the left circular dots are RCP4.5 (lower emissions scenario).

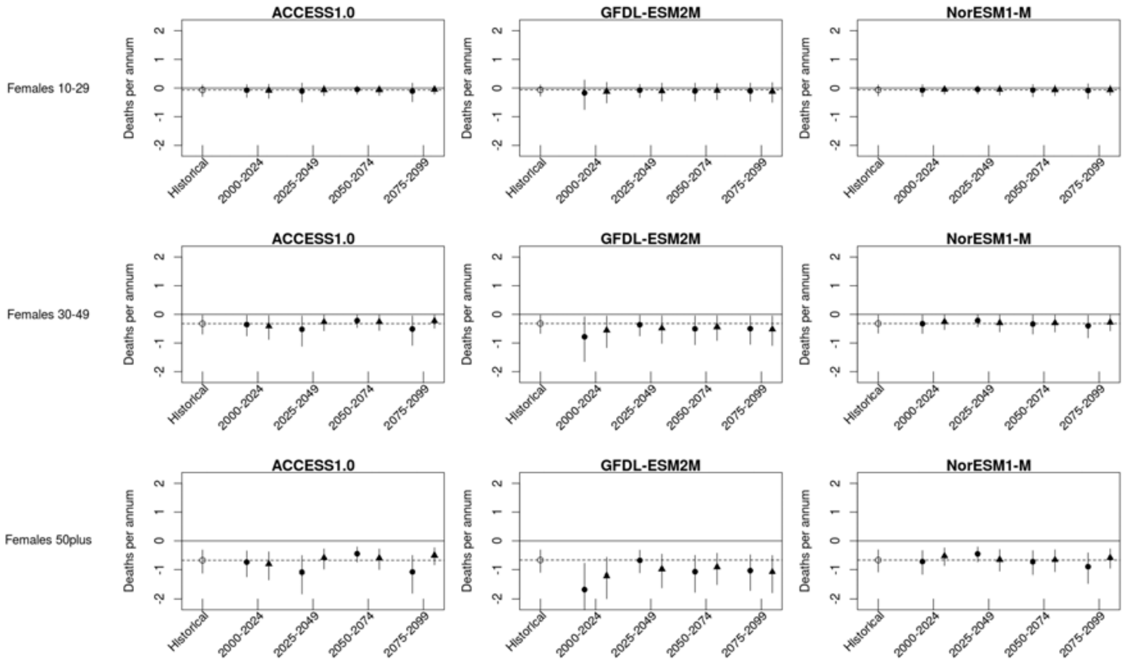

Figure S3: AN (and 95% CI) among females for quarter-centuries (25 year periods) compared to historical rates (1975–1999); the righthand triangular dots in each pair are RCP8.5 (high emissions scenario) whereas left circular dots are RCP4.5 (lower emissions scenario).

Percent changes in attributable numbers of suicides

Figure 3 of the main text shows the projected AN as deaths per annum during the two periods 1971–1999 (historical) and 2000–2099 (climate change scenarios). The estimated numbers are quite small. Thus, to better interpret how the excess drought-attributable suicides in full drought would be different in each scenario, we show percentage changes in excess attributable suicides in full drought compared to those in the historical period in Figure S4. In the Male subgroup aged 30–49 this shows an 84% (95%CI 2%, 159%) increase in excess suicides attributable to drought in the driest scenario (GFDL-ESM2M RCP4.5). Yet in the other subgroups, numbers of avoided suicides increased.

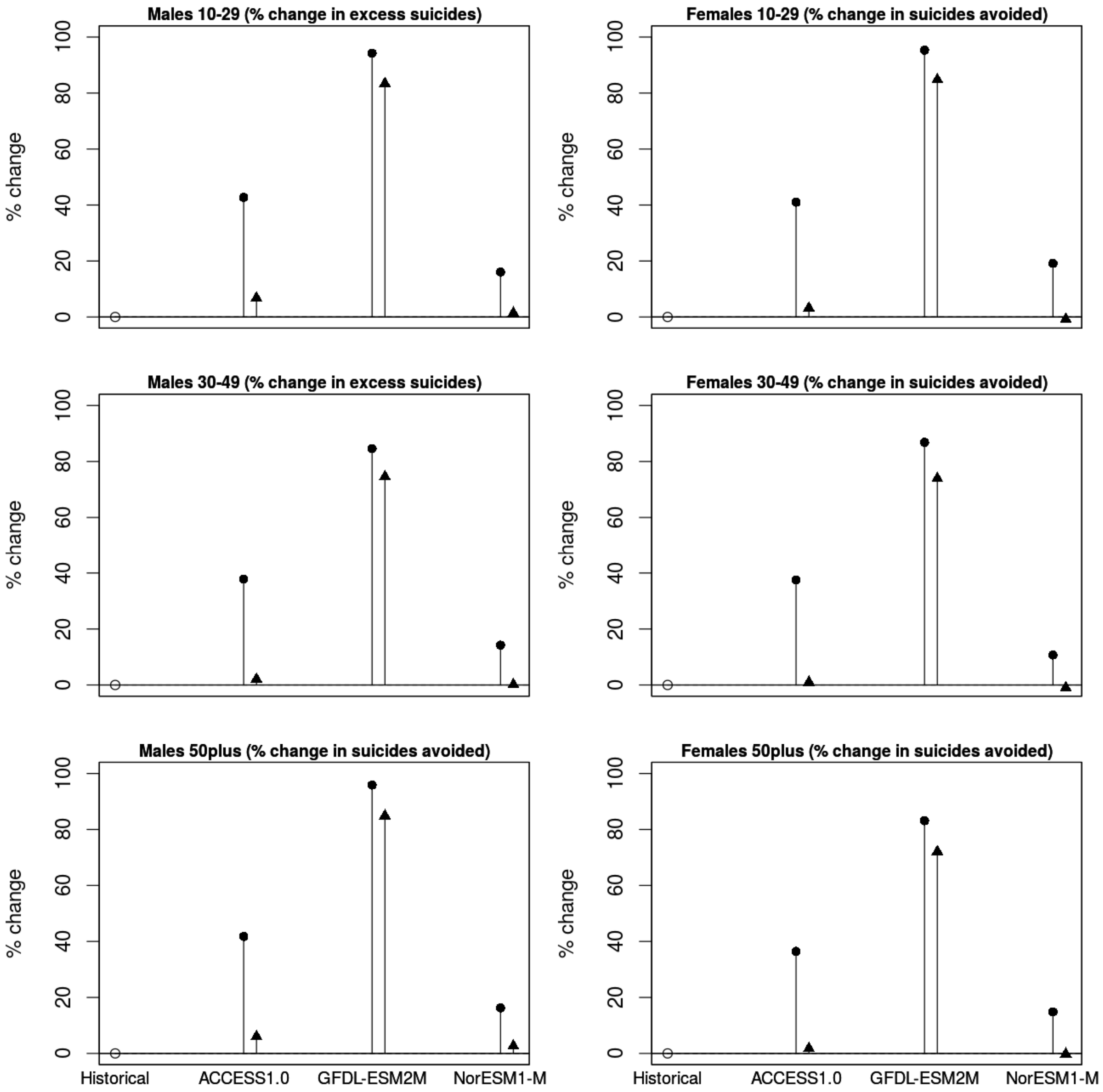

Figure S4: Percent changes in excess suicides attributable to full drought compared with the ANs in the historical period. The right-hand triangular dots in each pair are RCP8.5 (high emissions scenario) while the left circular dots are RCP4.5 (lower emissions scenario).

Suicide data

Deidentified unit records for each suicide (as determined by a coroner) in NSW between January 1970 and October 2007 were sourced from the Australian Causes of Death Unit Record File (Australian Bureau of Statistics, Causes of Death in Australia, Catalogue number: 3303.0, data available on request to the NSW coroner's office). The final months of 2007 were excluded because of the known delay in reporting of suicide deaths. Suicide records include the day of death, age, sex, and place of usual residence of the person who died. Unfortunately, exact locations of death and times living at the place of usual residence are not recorded in the mortality database, hindering precise exposure estimates.

ICD codes for suicide deaths, ICD-8, -9, and -10 classifications are considered comparable across this period (Australian Bureau of Statistics, 2000). Additionally, a long-term trend variable was included in the models to account for potential changes in the time series.

Population Data

Data were collected from the Australian census of population and housing, which is performed every 5 yrs. These data were available for local government areas during 1971–1981 and for statistical local areas during 1986–2006. We assigned each local area to its ABS Statistical Division (SD) region from the 2006 census edition. North and Far Western SDs were merged because the increased death counts better support multivariable log linear regression models.

The populations were categorized into the following age groups: 10–29, 30–49 and 50 plus. Populations of each age and sex group in each region were linearly interpolated by month prior to inclusion in models.

Hutchinson Drought Severity Index (HDSI)

This section describes changes to the HDSI method. In the original method the threshold for full drought was five months of relative dryness, not seven months as used here. This is because the original threshold was based on the historical rainfall records 1890–2008 and comparisons with government drought declarations data (Smith et al. 1992). In comparison, the present rolling 30-year benchmark period is introduced to support the use of climate change scenarios, as described below.

The original formulation of the HDSI used the longest historical period available as a benchmark (Smith et al. 1992). Previously, data from the entire century (1890–2008) was used as a benchmark to derive Hutchinson Scores (Hanigan et al. 2012). However, for the current use with the climate change scenarios data, the standard historical baseline was not appropriate because changes in climatic conditions over the future projections led to changes in relative percentile values for the historic period and poor comparability of drought indicators between scenarios. This necessitated the use of a rolling 30-yr look-back period so that drought indices for each year are based on percentiles derived using the previous 29 years alongside the nominated year.

The threshold for months in full drought, rather than mild drought, was revised upward to seven based on a comparison with the duration index computed for the 1890–2008 period, as described previously (Smith et al. 1992). A recursive partitioning tree model (RPART) was then used to classify the original HDSI index as a target variable and the new 30-year look-back drought index as a predictor. The results are shown in Figure S5 and indicate that a split at 6.25 is the optimal classification between dry and full drought.

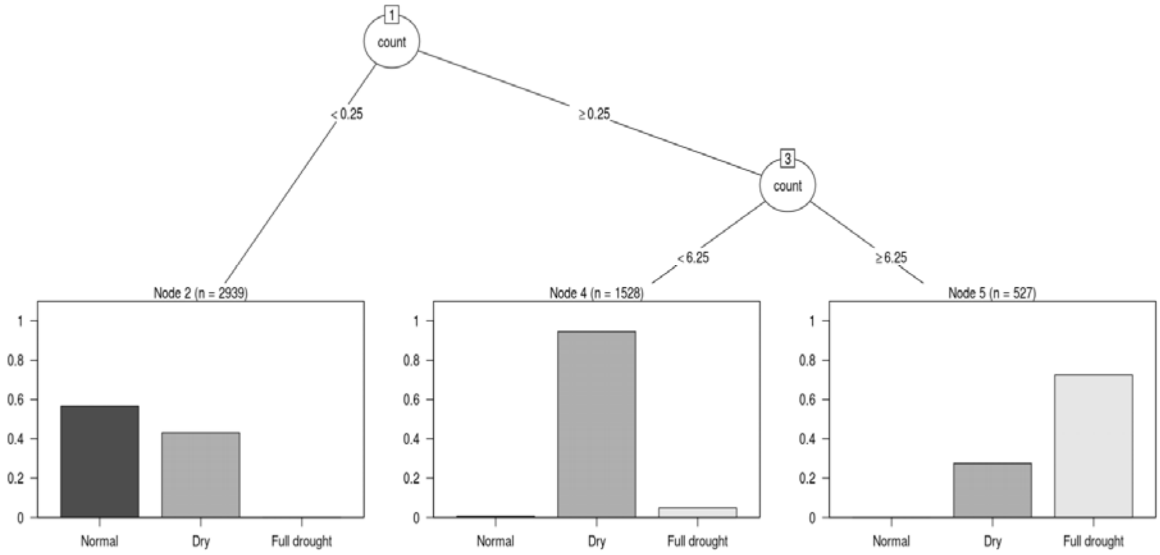

Figure S5: The new threshold for starting to count dry months as full drought was calculated using a recursive partitioning tree. The Hutchinson Drought Severity Index (HDSI) from the 1890–2008 baseline period was classified using the corresponding 30-year look-back HDSI estimate as a predictor. This showed that the best threshold was month 6.25 and over.

Climate Change scenario projections

Future droughts are likely to become longer and more severe. The extent of the changes that are expected depends on emission scenarios and on differences between the climate models used, with some predicting hotter, drier futures than others.

The two representative concentration pathways (RCP4.5 and RCP8.5) represent low- and high-emission scenarios, respectively. Data from three general circulation models (GCM) were chosen as a representative of plausible future climates predicted by the 40 models from Phase 5 of the Coupled Model Inter-comparison Project (CMIP5). The assessment by the CSIRO and the Bureau of Meteorology (2015) found that the ACCESS1.0 model had maximum consensus with the other models for many regions, the GFDL-ESM2M model was the hotter and drier model for many regions, and the NorESM1-M model was representative of a low warming, wetter scenario. The modelled projections are available through the CSIRO and Bureau of Meteorology, "Climate Change in Australia" dataset (https://doi.org/10.4225/08/55945F739A66D). These modelled data estimate rainfall by month between 2006 and 2100 as the percentage change compared to the 1986–2005 baseline climate.

GCM pixels were aggregated into SDs from the 2006 census. The GCMs were at the following resolutions: ACCESS1.0, 1.875 degrees EW × 1.25 degrees NS; NorESM1-M, 2.5 degrees EW × 1.85 degrees NS; and GFDL-ESM2M, 2.5 degrees EW × 2.0 degrees NS. Most SDs had several pixels overlapping their boundaries, thus limiting spatially variable changes in climatic conditions. For example, this approach assigns the same relative difference to places that are geographically separated by large distances and sometimes cross ecologically significant boundaries between arid interior regions and mountainous areas, or between inland and coastal zones.

Distributions of pixel values over subregions were summarized by calculating standard deviations for projected rainfall within each month from 2006 to 2099. The boxplots in Figures S6 and S7 show the range of spatial variability amongst the monthly climate projection data.

Maps of each month with maximum standard deviations within subregions (i.e. maximum spatial variability) for each GCM are shown in Figures S8, S9 and S10. Table S3 shows summary statistics for the spatial variability of monthly estimates. The median monthly deviations among pixels was less than 39 mm. The highest spatial variability of projected rainfall within subregions reflected extreme rainfall events in isolated small coastal regions of the north-east.

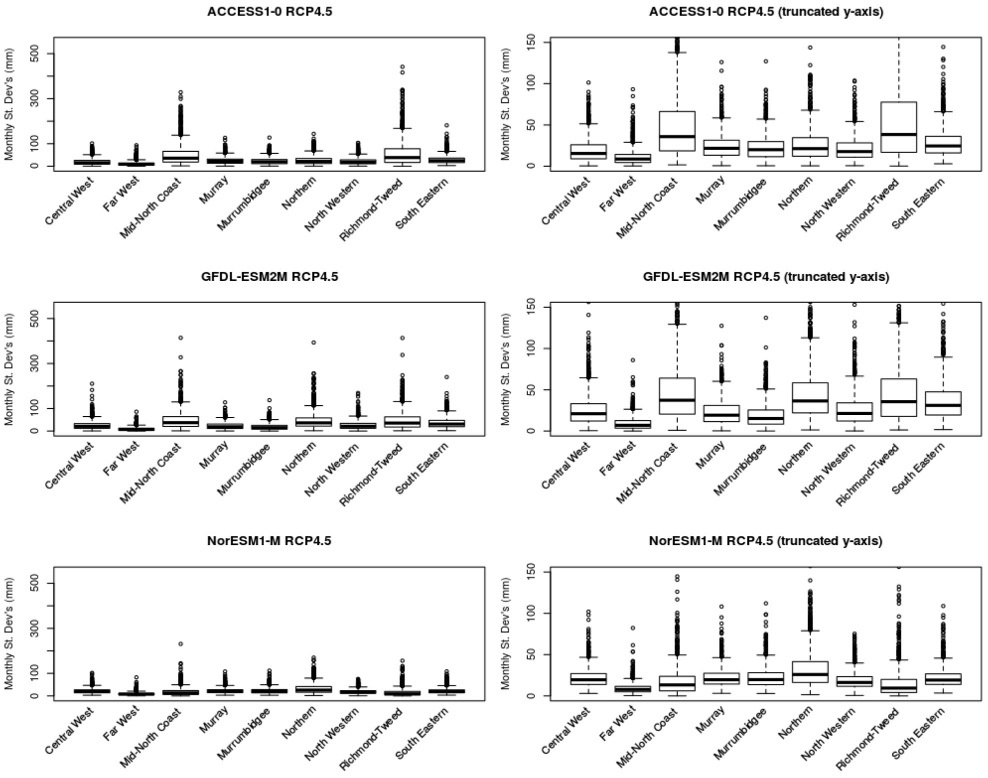

Figure S6: Boxplots of spatial variability (standard deviations among pixels) for each month of projected rainfall data in the RCP4.5 emissions scenarios.

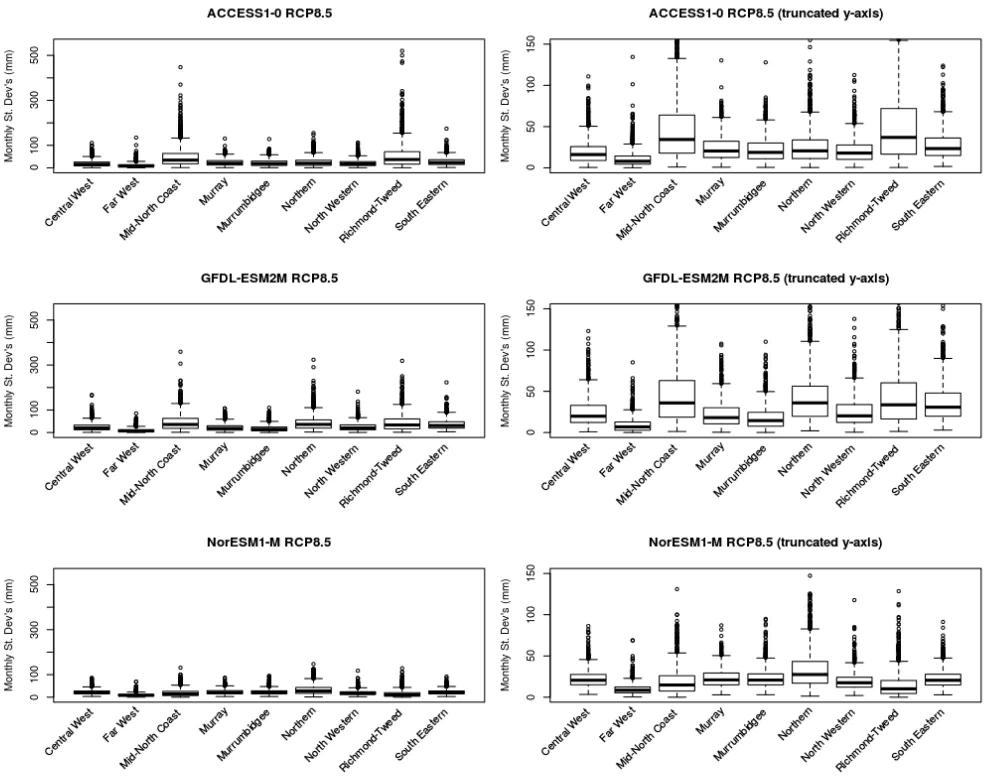

Figure S7: Boxplots of spatial variability (standard deviation among pixels) for each month of the projected rainfall data in RCP8.5 emissions scenarios

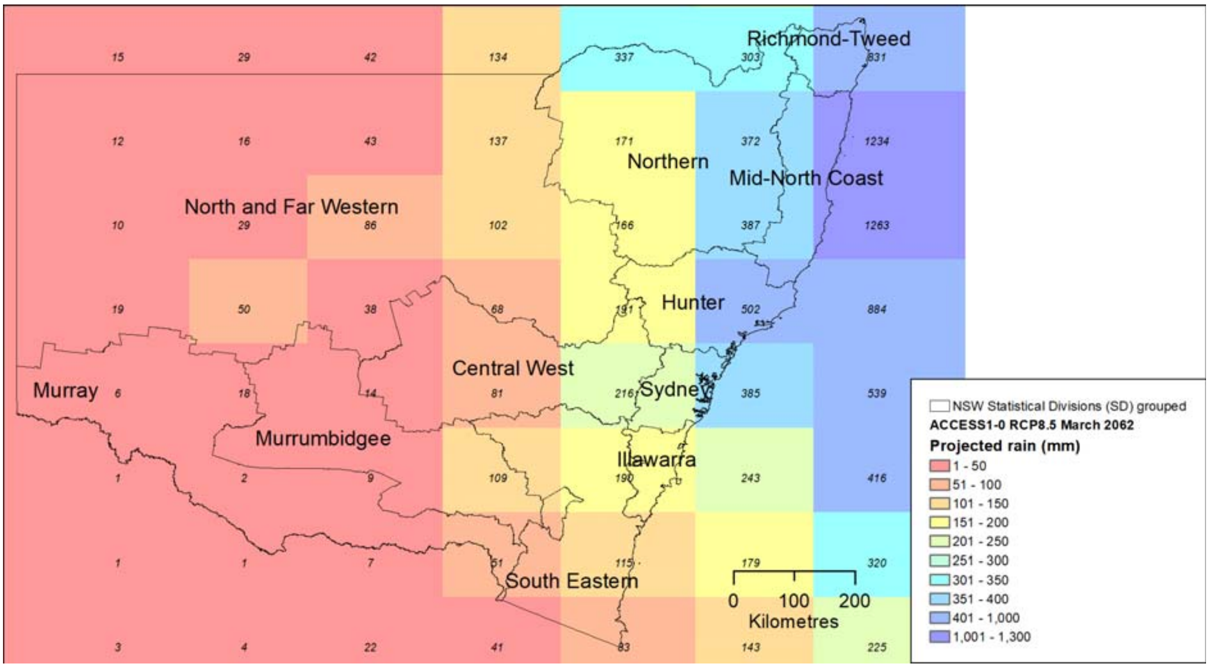

Figure S8: ACCESS1-0 global climate model (GCM) pixels during an event with high spatial variability within subregions

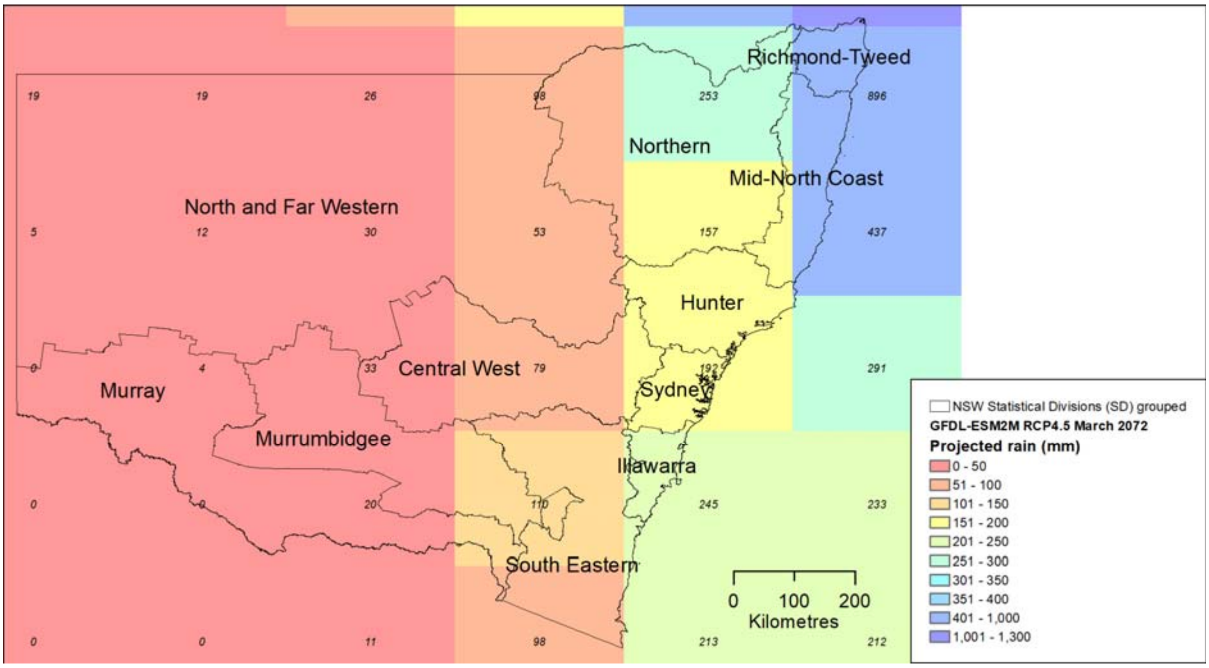

Figure S9: GFDL-ESM2M global climate model (GCM) pixels during an event with high spatial variability within subregions

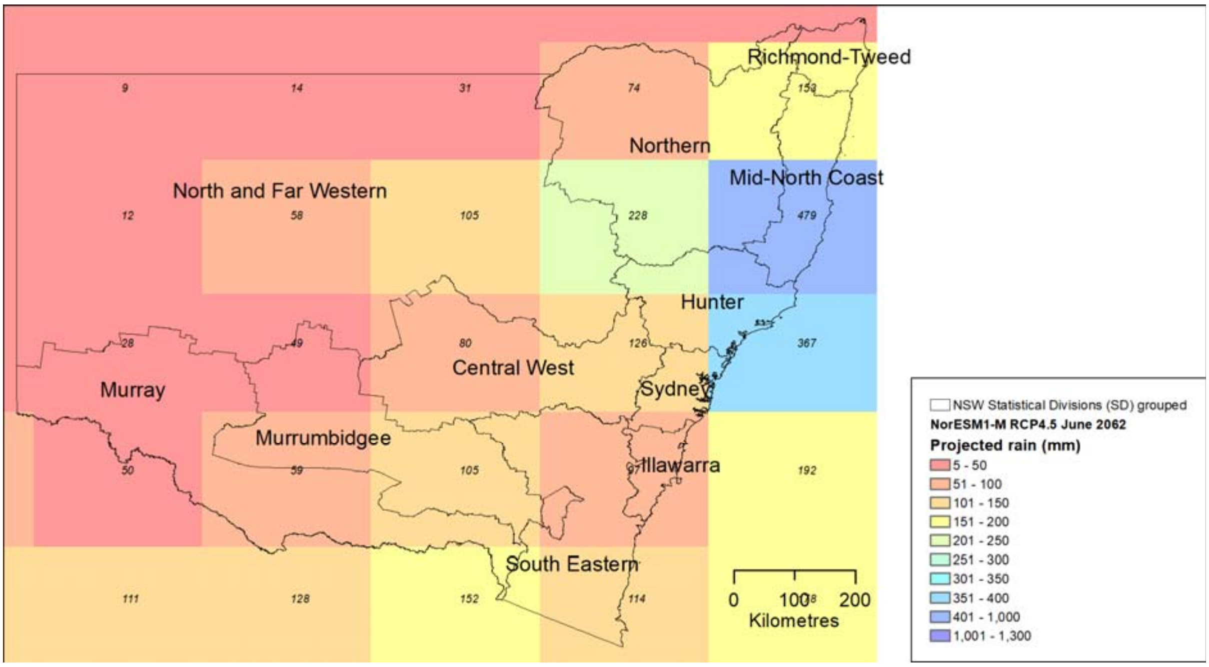

Figure S10: NorESM1-M global climate model (GCM) pixels during an event with high spatial variability within subregions

Table S3: Summary statistics of spatial variability (standard deviations among pixels) for each month of projected rainfall data

SD_NAME	GCM	RCP	Q1	Median	Q3
Central West	ACCESS1-0	RCP4.5	9	15	26
		RCP8.5	9	16	26
	GFDL-ESM2M	RCP4.5	12	21	33
		RCP8.5	12	20	33
	NorESM1-M	RCP4.5	14	19	27
		RCP8.5	15	21	28
Far West	ACCESS1-0	RCP4.5	5	9	14
		RCP8.5	4	8	14
	GFDL-ESM2M	RCP4.5	4	7	13
		RCP8.5	3	7	13
	NorESM1-M	RCP4.5	5	8	12
		RCP8.5	5	8	12
Mid-North Coast	ACCESS1-0	RCP4.5	19	36	66
		RCP8.5	18	34	64
	GFDL-ESM2M	RCP4.5	21	37	64
		RCP8.5	19	36	63
	NorESM1-M	RCP4.5	6	13	24
		RCP8.5	7	15	26
Murray	ACCESS1-0	RCP4.5	13	22	31
		RCP8.5	13	20	32
	GFDL-ESM2M	RCP4.5	11	19	31
		RCP8.5	11	18	30
	NorESM1-M	RCP4.5	14	20	27
		RCP8.5	15	21	29
Murrumbidgee	ACCESS1-0	RCP4.5	12	20	30
		RCP8.5	11	19	30
	GFDL-ESM2M	RCP4.5	8	15	25
		RCP8.5	8	14	25
	NorESM1-M	RCP4.5	14	20	28
		RCP8.5	15	21	28
Northern	ACCESS1-0	RCP4.5	12	21	35
		RCP8.5	11	21	34
	GFDL-ESM2M	RCP4.5	22	36	58
		RCP8.5	20	36	56
	NorESM1-M	RCP4.5	16	26	41
		RCP8.5	17	27	43
North Western	ACCESS1-0	RCP4.5	11	18	28
		RCP8.5	10	18	28
	GFDL-ESM2M	RCP4.5	12	21	34
		RCP8.5	12	20	34
	NorESM1-M	RCP4.5	12	16	23
		RCP8.5	12	17	24
Richmond-Tweed	ACCESS1-0	RCP4.5	17	38	78
		RCP8.5	17	37	72
	GFDL-ESM2M	RCP4.5	18	36	63
		RCP8.5	16	34	60
	NorESM1-M	RCP4.5	4	9	20
		RCP8.5	4	10	20
South Eastern	ACCESS1-0	RCP4.5	16	25	36
		RCP8.5	15	23	36
	GFDL-ESM2M	RCP4.5	19	31	47
		RCP8.5	20	31	48
	NorESM1-M	RCP4.5	14	19	27
		RCP8.5	15	21	28

Final model parameters for all covariates

Parameters estimated from this model are shown in Table S4.

Table S4: Parameters estimated for the model of historical drought and suicide data

	Estimate	Std. Error	z value	Pr(>|z|)
(Intercept)	-11.654313	0.0790571	-147.41642	0
DrtMales10_29rural	0.0477414	0.0295608	1.615024	0.1063055
DrtMales30_49rural	0.096151	0.0244134	3.9384486	8.20E-05
DrtMales50plusrural	-0.0072455	0.0269394	-0.2689541	0.787965
DrtFemales10_29rural	-0.0401956	0.0650093	-0.6183061	0.5363736
DrtFemales30_49rural	-0.1129361	0.0520602	-2.1693368	0.0300571
DrtFemales50plusrural	-0.2207487	0.0525736	-4.1988475	2.68E-05
ns(DrtMales10_29urban, df = 6)1	0	0	NA	NA
ns(DrtMales10_29urban, df = 6)2	0	0	NA	NA
ns(DrtMales10_29urban, df = 6)3	0	0	NA	NA
ns(DrtMales10_29urban, df = 6)4	0	0	NA	NA
ns(DrtMales10_29urban, df = 6)5	0	0	NA	NA
ns(DrtMales10_29urban, df = 6)6	0.0011556	0.059263	0.0194995	0.9844426
DrtMales30_49urban	-0.0259964	0.0146335	-1.7765052	0.0756497
ns(DrtMales50plusurban, df = 4)1	0.0961064	0.0571638	1.681245	0.0927153
ns(DrtMales50plusurban, df = 4)2	0	0	NA	NA
ns(DrtMales50plusurban, df = 4)3	0	0	NA	NA
ns(DrtMales50plusurban, df = 4)4	0	0	NA	NA
DrtFemales10_29urban	0.058868	0.0326339	1.8038908	0.0712484
ns(DrtFemales30_49urban, df = 3)1	0	0	NA	NA
ns(DrtFemales30_49urban, df = 3)2	0	0	NA	NA
ns(DrtFemales30_49urban, df = 3)3	0.0234058	0.0650414	0.3598592	0.7189524
DrtFemales50plusurban	-0.0044733	0.0246977	-0.181121	0.8562726
agegp230_49	0.895925	0.0731212	12.252603	1.63E-34
agegp250plus	1.0525728	0.0735452	14.311925	1.84E-46
rural	0.0274924	0.0454512	0.6048767	0.5452609
sd_groupHunter	0	0	NA	NA
sd_groupIllawarra	0.0386792	0.0347822	1.1120409	0.2661206
sd_groupMid-North Coast	-0.0564936	0.0510655	-1.1062984	0.2685974
sd_groupMurray	0.0239014	0.0609314	0.3922672	0.6948608
sd_groupMurrumbidgee	0.0249126	0.0557265	0.4470513	0.654838
sd_groupNorth and Far Western	0.1941735	0.0536107	3.62192	0.0002924
sd_groupNorthern	0.0757917	0.0522459	1.4506726	0.146871
sd_groupRichmond-Tweed	0.0864046	0.0527123	1.6391714	0.1011776
sd_groupSouth Eastern	0.0548102	0.0533753	1.0268834	0.3044754
sd_groupSydney	0.0673497	0.0234876	2.8674582	0.0041378
sex2	-0.61975	0.1059403	-5.8499948	4.92E-09
tmax_anomaly	0.0161442	0.0052987	3.0468428	0.0023126
ns(time, 3)1	1.0015687	0.0580024	17.26771	8.23E-67
ns(time, 3)2	0.1072501	0.1396693	0.7678857	0.4425551
ns(time, 3)3	-0.4921425	0.0570039	-8.6334901	5.95E-18
agegp230_49:sex2	0.138544	0.132523	1.0454336	0.2958226
agegp250plus:sex2	-0.0652762	0.1319496	-0.4947053	0.6208082
agegp230_49:ns(time, 3)1	-0.6036243	0.0759658	-7.946004	1.93E-15
agegp250plus:ns(time, 3)1	-0.9071519	0.0786734	-11.530607	9.25E-31
agegp230_49:ns(time, 3)2	-0.8404583	0.1792178	-4.6895918	2.74E-06
agegp250plus:ns(time, 3)2	-1.1544727	0.1813407	-6.3663185	1.94E-10
agegp230_49:ns(time, 3)3	0.3715438	0.069999	5.3078465	1.11E-07
agegp250plus:ns(time, 3)3	-0.1451594	0.0730854	-1.9861611	0.0470154
sex2:ns(time, 3)1	-0.8939064	0.1311295	-6.8169767	9.30E-12
sex2:ns(time, 3)2	-1.6006529	0.2721127	-5.8823151	4.05E-09
sex2:ns(time, 3)3	-0.0190852	0.1236775	-0.1543142	0.877362
agegp230_49:sex2:ns(time, 3)1	0.2095445	0.1658932	1.2631286	0.206543
agegp250plus:sex2:ns(time, 3)1	0.2230522	0.1664532	1.3400298	0.1802357
agegp230_49:sex2:ns(time, 3)2	0.1066869	0.3413548	0.3125396	0.7546305
agegp250plus:sex2:ns(time, 3)2	0.8682599	0.3392836	2.5590977	0.0104944
agegp230_49:sex2:ns(time, 3)3	-0.5910189	0.1531063	-3.8601868	0.0001133
agegp250plus:sex2:ns(time, 3)3	-0.2782652	0.153384	-1.8141734	0.069651

Using sine and cosine terms to capture seasonality

Instead of a cyclic spline, sine and cosine terms were used to capture seasonality. The resulting parameters were not meaningfully different in the sensitivity test model.

Table S5: Parameters estimated for the model of historical data using a sine and cosine seasonality function and only linear functions for drought and suicide

	Estimate	Std. Error	z value	Pr(>|z|)
(Intercept)	-11.597965	0.0609543	-190.27318	0
sin(timevar * 2 * pi)	-0.023362	0.0090412	-2.5839669	0.0097671
cos(timevar * 2 * pi)	0.0361799	0.0090763	3.9862099	6.71E-05
DrtMales10_29rural	0.0474916	0.0295611	1.6065565	0.1081517
DrtMales30_49rural	0.0958622	0.0244145	3.926451	8.62E-05
DrtMales50plusrural	-0.0075024	0.0269402	-0.2784832	0.7806415
DrtFemales10_29rural	-0.040446	0.0650098	-0.6221517	0.5338421
DrtFemales30_49rural	-0.113221	0.052061	-2.174776	0.0296469
DrtFemales50plusrural	-0.2209808	0.0525743	-4.2032123	2.63E-05
DrtMales10_29urban	0.0001496	0.0165715	0.0090283	0.9927965
DrtMales30_49urban	-0.0261699	0.0146355	-1.7881112	0.0737581
DrtMales50plusurban	-0.0270403	0.0159847	-1.6916324	0.0907161
DrtFemales10_29urban	0.0586858	0.0326359	1.7982011	0.0721452
DrtFemales30_49urban	0.0089711	0.0254842	0.3520251	0.7248194
DrtFemales50plusurban	-0.0046462	0.0246992	-0.1881095	0.8507908
agegp230_49	0.8959776	0.0731277	12.252238	1.63E-34
agegp250plus	1.0526491	0.0735517	14.311695	1.85E-46
rural	0.0275513	0.0454515	0.6061696	0.5444022
sd_groupHunter	0	0	NA	NA
sd_groupIllawarra	0.0386937	0.0347821	1.1124576	0.2659414
sd_groupMid-North Coast	-0.0564765	0.0510654	-1.1059633	0.2687424
sd_groupMurray	0.0238961	0.0609314	0.3921796	0.6949255
sd_groupMurrumbidgee	0.0249044	0.0557265	0.4469045	0.654944
sd_groupNorth and Far Western	0.1941612	0.0536107	3.621688	0.0002927
sd_groupNorthern	0.0757532	0.0522459	1.4499353	0.1470766
sd_groupRichmond-Tweed	0.0864454	0.0527123	1.6399475	0.1010161
sd_groupSouth Eastern	0.0548168	0.0533753	1.0270067	0.3044173
sd_groupSydney	0.0673636	0.0234876	2.8680493	0.0041301
sex2	-0.6196633	0.1059509	-5.8485866	4.96E-09
tmax_anomaly	0.0162727	0.0052974	3.0718219	0.0021276
ns(time, 3)1	1.0014746	0.0580024	17.266076	8.47E-67
ns(time, 3)2	0.1064021	0.1396791	0.7617608	0.4462028
ns(time, 3)3	-0.492332	0.0570045	-8.6367252	5.78E-18
agegp230_49:sex2	0.1385546	0.1325369	1.0454041	0.2958362
agegp250plus:sex2	-0.0653136	0.131963	-0.4949386	0.6206435
agegp230_49:ns(time, 3)1	-0.6036338	0.0759655	-7.9461545	1.92E-15
agegp250plus:ns(time, 3)1	-0.9071125	0.0786731	-11.530142	9.30E-31
agegp230_49:ns(time, 3)2	-0.8405461	0.1792303	-4.6897543	2.74E-06
agegp250plus:ns(time, 3)2	-1.1546478	0.181353	-6.3668526	1.93E-10
agegp230_49:ns(time, 3)3	0.3715233	0.0699997	5.3074958	1.11E-07
agegp250plus:ns(time, 3)3	-0.1451472	0.0730862	-1.9859725	0.0470364
sex2:ns(time, 3)1	-0.893885	0.1311292	-6.8168283	9.31E-12
sex2:ns(time, 3)2	-1.6008748	0.2721315	-5.8827256	4.04E-09
sex2:ns(time, 3)3	-0.0190625	0.123679	-0.1541291	0.877508
agegp230_49:sex2:ns(time, 3)1	0.2096026	0.1658929	1.2634811	0.2064163
agegp250plus:sex2:ns(time, 3)1	0.2230874	0.1664527	1.3402453	0.1801656
agegp230_49:sex2:ns(time, 3)2	0.1066412	0.3413799	0.3123829	0.7547496
agegp250plus:sex2:ns(time, 3)2	0.8683166	0.3393072	2.5590868	0.0104948
agegp230_49:sex2:ns(time, 3)3	-0.5910278	0.1531083	-3.8601953	0.0001133
agegp250plus:sex2:ns(time, 3)3	-0.2782768	0.1533859	-1.8142263	0.0696429

NB: “timevar” is used for the sinusoidal function and is created as: index/ (length(index) / (length(index)/12)), where the index represents a sequence from the first time to the last time.

Sensitivity testing for the splines of time

We ran sensitivity analyses that increased the df allocated to both trend (Time) and seasonality (Month) spline terms. Initially, the df were increased for the spline of Time while keeping the seasonal term constant df = 4, as in the main model. Subsequent increases in df for the Month spline were tested with a constant df = 3 for Time. Finally, df were increased on both splines simultaneously, and the models were refitted sequentially to compare the estimated RRs for each age-sex-region subgroup. To allow several peaks and troughs through the annual cycle, the df on the trend were increased from 3 to 25 (or 1.5 df per year of data) and from 3 to 7 for the seasonality term. The results are shown in Figure S11 and these additional analyses did not change the effect estimates reported in the main models.

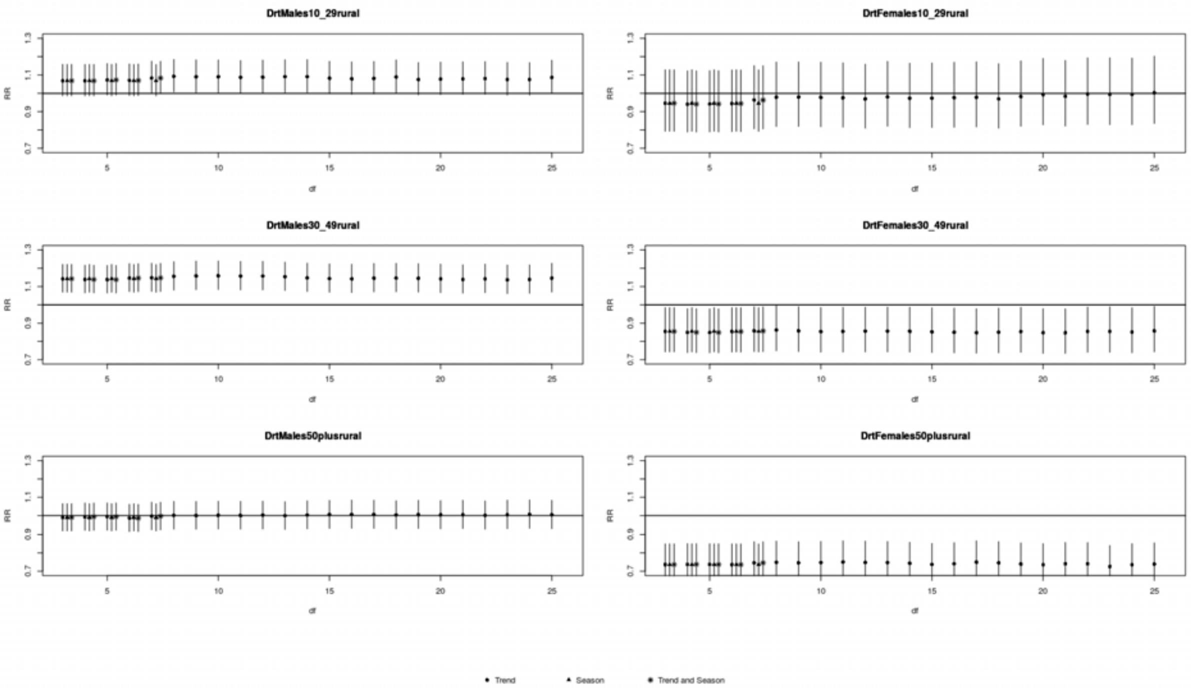

Figure S11: Sensitivity analyses with increased df allocated to trend (Time) and seasonality (Month) spline terms. First, the df for the spline of Time was incrementally increased while keeping the seasonal term constant at df = 4 (RRs shown as circles). Second, the df for the spline on Month were increased while keeping the spline of Time constant df = 3 (RRs shown as triangles). Third, the df on both splines were increased simultaneously (only up to df = 7 (RRs shown as asterisks).

Sensitivity test for aggregating all rural SDs

To address small counts and many zeros, we increased the scale of the death counts by aggregating records across the entire rural section of the state of NSW and averaging the drought variable across all rural SDs in each month. Although this resulted in smoothing of the variance in exposure and therefore attenuated the drought risk estimate (due to inclusion of unaffected areas), these sensitivity analyses showed similar associations of suicide rates with drought durations, suggesting that the model is robust.

Sensitivity test for zero-inflated Poisson model

To account for possible over-dispersion, a final sensitivity analysis was performed using a zero-inflated Poisson GAM. This model assumes two distributions, one where the count is always zero and another were the counts have a Poisson distribution. This analysis also broadly agreed with the main models, but required additional modelling assumptions. Hence, the Poisson model is more parsimonious and preferable, and was kept for the main analyses.

References

Australian Bureau of Statistics. *Suicides 1921–1998*. (Australian Bureau of Statistics, Canberra, Australia., 2000).

CSIRO and Bureau of Meteorology. (2015). Chapter 9 Using climate change data in impact assessment and adaptation planning in Climate Change in Australia Information for Australia's Natural Resource Management Regions: Technical Report. Canberra, Australia.' Retrieved from https://www.climatechangeinaustralia.gov.au/media/ccia/2.1.6/cms_page_media/168/CCIA_2015_NRM_TR_Chapter%209.pdf on 13/04/2019.

Durkheim E (1951) Suicide: A study in sociology (English translation 1951, first edition in French 1897). New York, NY: Simon and Schuster.

Hanigan, I. C., Butler, C. D., Kokic, P. N. & Hutchinson, M. F. Suicide and drought in New South Wales, Australia, 1970-2007. *Proceedings of the National Academy of Sciences of the United States of America* **109**, 13950–13955 (2012).

Hanigan, I.C., Guillaume, J., Markham, F., Williamson, G., Szarka, I., Hanson, J. O, Sparks, A. H. (2016) AWAPTOOLS: Software to download and reformat Australian Water Availability Grids from BoM/CSIRO. https://github.com/swish-climate-impact-assessment/awaptools

Jones DA, Wang W, Fawcett R 2009 'High-quality spatial climate data-sets for Australia.' Australian Meteorological and Oceanographic Journal 58, 233-248.

Mullins JT and White C (2019) Temperature and mental health: Evidence from the spectrum of mental health outcomes, J. Health Econ., vol. 68, p. 102240.

Smith, D. I., Hutchinson, M. F. & McArthur, R. J. *Climatic and Agricultural Drought: Payments and Policy*. (Australian National University, 1992).

Climate change scenario data sources

ACCESS1.0, Maximum consensus for many regions

RCP4.5

http://dapds00.nci.org.au/thredds/fileServer/ua6_4/CMIP5/derived/CMIP5/GCM/native/CSIRO-BOM/ACCESS1-0/rcp45/mon/atmos/Amon/r1i1p1/v20170704/pr/seassum-timeseries-percent-change/pr_Amon_ACCESS1-0_rcp45_r1i1p1_2006-2100-percent-change-wrt-1986-2005-seassum-timeseries_native.nc

RCP8.5

http://dapds00.nci.org.au/thredds/fileServer/ua6_4/CMIP5/derived/CMIP5/GCM/native/CSIRO-BOM/ACCESS1-0/rcp85/mon/atmos/Amon/r1i1p1/v20170704/pr/seassum-timeseries-percent-change/pr_Amon_ACCESS1-0_rcp85_r1i1p1_2006-2100-percent-change-wrt-1986-2005-seassum-timeseries_native.nc

NorESM1-M, Low warming wettest representative model

RCP4.5

http://dapds00.nci.org.au/thredds/fileServer/ua6_4/CMIP5/derived/CMIP5/GCM/native/NCC/NorESM1-M/rcp45/mon/atmos/Amon/r1i1p1/v20170704/pr/seassum-timeseries-percent-change/pr_Amon_NorESM1-M_rcp45_r1i1p1_2006-2100-percent-change-wrt-1986-2005-seassum-timeseries_native.nc

RCP8.5

http://dapds00.nci.org.au/thredds/fileServer/ua6_4/CMIP5/derived/CMIP5/GCM/native/NCC/NorESM1-M/rcp85/mon/atmos/Amon/r1i1p1/v20170704/pr/seassum-timeseries-percent-change/pr_Amon_NorESM1-M_rcp85_r1i1p1_2006-2100-percent-change-wrt-1986-2005-seassum-timeseries_native.nc

GFDL-ESM2M, Hotter and drier model for many clusters

RCP4.5

http://dapds00.nci.org.au/thredds/fileServer/ua6_4/CMIP5/derived/CMIP5/GCM/native/NOAA-GFDL/GFDL-ESM2M/rcp45/mon/atmos/Amon/r1i1p1/v20170704/pr/seassum-timeseries-percent-change/pr_Amon_GFDL-ESM2M_rcp45_r1i1p1_2006-2100-percent-change-wrt-1986-2005-seassum-timeseries_native.nc

RCP8.5

http://dapds00.nci.org.au/thredds/fileServer/ua6_4/CMIP5/derived/CMIP5/GCM/native/NOAA-GFDL/GFDL-ESM2M/rcp85/mon/atmos/Amon/r1i1p1/v20170704/pr/seassum-timeseries-percent-change/pr_Amon_GFDL-ESM2M_rcp85_r1i1p1_2006-2100-percent-change-wrt-1986-2005-seassum-timeseries_native.nc
